# AlphaFold‐Guided Bespoke Gene Editing Enhances Field‐Grown Soybean Oil Contents

**DOI:** 10.1002/advs.202500290

**Published:** 2025-05-14

**Authors:** Jie Wang, Li Zhang, Shoudong Wang, Xin Wang, Suning Li, Pingping Gong, Mengyan Bai, Arnav Paul, Nathan Tvedt, Hengrui Ren, Maoxiang Yang, Zhihui Zhang, Shaodong Zhou, Jiayi Sun, Xianjin Wu, Huaqin Kuang, Zhenghua Du, Yonghui Dong, Xiaolei Shi, Meina Li, Diwakar Shukla, Long Yan, Yuefeng Guan

**Affiliations:** ^1^ College of Resources and Environment Fujian Agriculture and Forestry University Fuzhou 350002 China; ^2^ Guangdong Provincial Key Laboratory of Plant Adaptation and Molecular Design Innovative Center of Molecular Genetics and Evolution School of Life Sciences Guangzhou University Guangzhou 510006 China; ^3^ Key Laboratory of Soybean Molecular Design Breeding State Key Laboratory of Black Soils Conservation and Utilization Northeast Institute of Geography and Agroecology Chinese Academy of Sciences Changchun 130102 China; ^4^ Jiangxi Province Key Laboratory of Oil Crops Genetic Improvement Crop Institute Jiangxi Academy of Agricultural Sciences Nanchang 330200 China; ^5^ Fujian Provincial Key Laboratory of Haixia Applied Plant Systems Biology Haixia Institute of Science and Technology and School of Life Sciences Fujian Agriculture and Forestry University Fuzhou 350002 China; ^6^ Department of Chemistry University of Illinois Urbana‐Champaign Urbana IL 61801 USA; ^7^ Center for Biophysics and Quantitative Biology University of Illinois Urbana‐Champaign Urbana IL 61801 USA; ^8^ Hebei Laboratory of Crop Genetics and Breeding Institute of Cereal and Oil Crops Hebei Academy of Agricultural and Forestry Sciences Shijiazhuang 050035 China; ^9^ Department of Chemical and Biomolecular Engineering University of Illinois Urbana‐Champaign Urbana IL 61801 USA

**Keywords:** AlphaFold, CRISPR–Cas9, gene editing, oil content, precision breeding, soybean, SWEET transporter

## Abstract

Enhancing the oil or protein content of soybean, a major crop for oil and protein production is highly desirable. *GmSWEET10a* encodes a sugar transporter that is strongly selected during domestication and breeding, increasing seed size and oil content. GmSWEET10b is functionally similar to GmSWEET10a, yet has not been artificially selected. Here, AlphaFold is used to find that C‐terminal variants of GmSWEET10a can endow enhanced or reduced transport activity. Guided by AlphaFold, the functionality is improved for GmSWEET10a in terms of oil content through gene editing. Furthermore, novel *GmSWEET10b* haplotypes possessing strengthened or weakened sugar‐transport capabilities that are absent in nature are engineered. Consequently, soybean oil content or protein content in independent *GmSWEET10b* gene‐edited lines during multi‐year and multi‐site field trials is consistently increased, without negatively affecting yield. The study demonstrates that the combination of AlphaFold‐guided protein design and gene editing has the potential to generate novel beneficial alleles, which can optimize protein function in the context of crop breeding.

## Graphical abstract

An AlphaFold‐guided method is developed to functionally optimize soybean sugar transporters and achieve bespoke gene editing of *GmSWEET10a/b* to improve oil content in an elite soybean cultivar in multi‐year, multi‐site field trials. The combination of AI‐guided protein design and gene editing may unlock a huge potential to improve the genetic trait *de novo*.

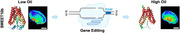

## 1 Introduction

Enhancing the function of proteins affecting important traits is desirable in crop breeding toward meeting the demands of 9.8 billion people by 2050. CRISPR–Cas‐based gene editing has greatly expanded the scope of the potential genetic variation space in crop improvement. So far, CRISPR–Cas is mainly used for knocking out unfavorable genes, modulating expression by promoter editing, or in precision editing by base editing or prime editing.^[^ ^1^, ^2^ ^]^ However, the predictive artificial evolution of functional proteins actioned by gene editing, particularly for genes without knowledge of beneficial haplotypes, remains a challenge. Protein‐structure‐prediction artificial intelligence technologies such as AlphaFold have shown remarkable success.^[^ ^3^ ^]^ Guided by AlphaFold‐predicted structures, new proteins can be characterized, optimized, or designed.^[^ ^4^ ^]^ In precision crop breeding, a combination of AlphaFold‐assisted protein design and gene editing has the potential to optimize or design proteins, although such a concept has not yet been demonstrated experimentally.^[^ ^5^ ^]^

The legume crop soybean (*Glycine max* (L.) Merr.) supplies oil and protein for food and feed, and with over 348 million tons harvested in 2022 alone (www.fao.org/faostat) accounts for 60% of global oilseed production.^[^ ^6^ ^]^ Oil and protein content has been a key trait subject to intense artificial selection in modern breeding, and further improvement of oil/protein content remains desirable to feed a growing population.^[^ ^7^ ^]^ It is widely known that there is a trade‐off between seed oil and protein content in soybeans. Efforts to improve soybean oil or protein content tended to focus on the identification and exploitation of beneficial natural alleles that have been artificially selected.^[^ ^7^, ^8^ ^]^ Nevertheless, many beneficial alleles are already fixed within elite high‐oil or high‐protein germplasm, placing a ceiling on further improvement. Besides, for the majority of the functional trait genes, favorable genetic variation may not evolve naturally, also bottlenecking further meaningful agronomic improvement.^[^ ^9^ ^]^

*GmSWEET10a* encodes a Sugars Will Eventually be Exported Transporters (SWEETs)‐a family sugar transporter that facilitates sugar allocation into the embryo and enhances fatty‐acid biosynthesis.^[^ ^10^, ^11^, ^12^ ^]^ During domestication and breeding, *GmSWEET10a* was extensively selected, conferring simultaneous increases in seed size and oil contents.^[^ ^10^, ^11^, ^12^ ^]^ *GmSWEET10b* is functionally redundant with *GmSWEET10a*, rendering it a potential target for rational improvement of oil content and seed size. However, *GmSWEET10b* was typically not artificially selected during domestication.^[^ ^11^ ^]^ Single and double *GmSWEET10a/b* knock‐out mutants developed by gene editing have less oil, smaller seeds, and yield penalties,^[^ ^11^ ^]^ rendering them unsuitable for agronomic use. Here, aided by AlphaFold‐predicted protein structures, we precisely designed bespoke, superior GmSWEET10b haplotypes via artificial evolution and consistently improved soybean oil content in field trials across multiple locations and seasons.

## 2 Result

### 2.1 The C‐Terminus Variations of GmSWEET10a Determine Soybean Oil Content

**Figure 1:**
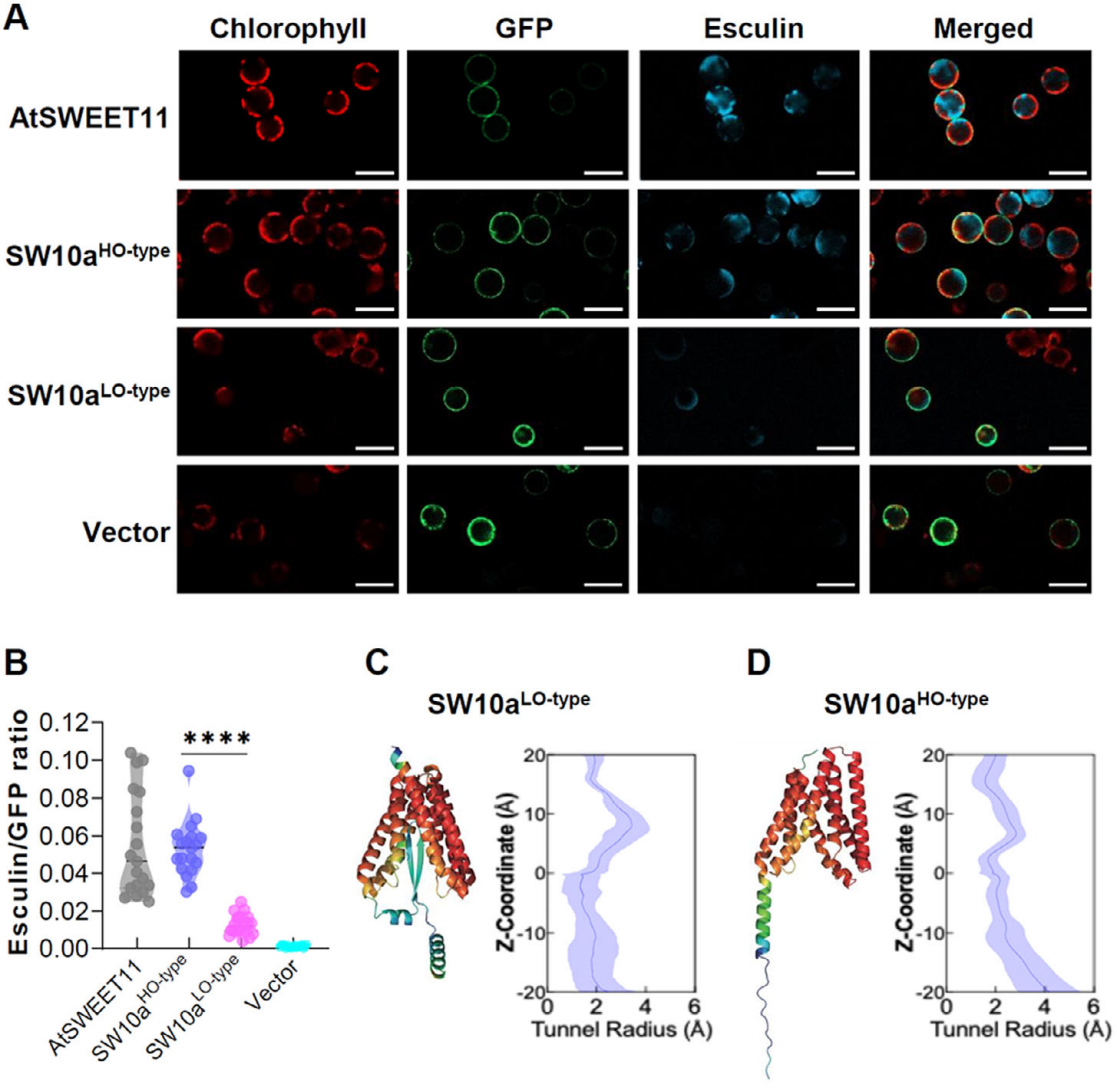
HO‐type GmSWEET10a has a higher likelihood of facilitating sugar transport. A) Representative images of Arabidopsis protoplasts transformed with *AtSWEET11*, *GmSWEET10a* HO‐type, and LO‐type genes co‐expressed with *GFP*, and the empty‐vector control. GFP fluorescence is shown in green, esculin fluorescence in cyan, and chlorophyll autofluorescence in red. Scale bar = 50 µm. B) Normalization of SWEET transport activity by the ratio of esculin fluorescence intensity in the vacuole of protoplasts versus GFP fluorescence in the plasma membrane. Fluorescence intensities were normalized to the radius of the respective protoplast under the assumption of a spherical shape (n ≥ 19). Each dot represents an independent measurement. Statistically significant differences are indicated by different asterisks (^****^ indicates *p* < 0.0001, one‐way ANOVA with Kruskal–Wallis test). C,D) AlphaFold2‐based protein‐haplotype‐structure and tunnel profile of HO‐type C) and LO‐type D) GmSWEET10a. The tunnel profile for LO‐type shows a closed substrate‐translocation pore in the lower half, and HO‐type shows an open substrate‐translocation pore in the lower half. Shading shows the standard deviation of the tunnel profile.

A 2‐nt indel variant (“CC–” or “CC+”) is present in *GmSWEET10a* across diverse soybean germplasm and causes a frameshift in the C‐terminus region.^[^ ^10^, ^11^, ^12^ ^]^ The CC– allele is strongly associated with higher oil contents and the CC+ haplotype is found predominantly in low‐oil germplasm (Figure S1, Supporting Information). The CC– haplotype was designated as the high‐oil type (“HO‐type,” associated with higher oil), and the CC+ haplotype as the low‐oil type (“LO‐type,” associated with lower oil, Figure S1, Supporting Information). We used Arabidopsis mesophyll protoplasts to quantify sucrose‐transport activities with the fluorescent sucrose analog esculin.^[^ ^13^ ^]^ Compared to the AtSWEET11 positive control, protoplasts transformed with HO‐type *GmSWEET10a* accumulated similar esculin levels in their vacuoles, indicating comparable and functional sucrose‐transport activity. In contrast, LO‐type GmSWEET10a showed significantly lower apparent transporter activity than the HO‐type GmSWEET10a (**Figure** **1**A,B).

We performed CRISPR–Cas9 targeting the C‐terminus of HO‐type GmSWEET10a in the elite cultivar HC6, and identified two homozygous mutant alleles designated *sw10a‐lo‐1* (+1 bp) and *sw10a‐lo‐2* (–2 bp) (Figure S2, Supporting Information). *GmSWEET10a* expression was unaffected or slightly increased, but both *lo* mutants had lower oil content (18.43–19.57% vs 20.33% in HC6) and smaller seeds than c.v. HC6 controls (Figures S2 and S3, Supporting Information). These results validated that variation at the C‐terminus affects the functionality and transport activity of GmSWEET10a. Nevertheless, the C‐terminus of SWEETs, attributed to their often‐unobserved flexible residues and intrinsically disordered nature, remains an elusive structural element.^[^ ^14^, ^15^ ^]^ In addition, *GmSWEET10a/10b* expression is confined to the thick‐walled parenchyma cell layer of the seed coat, hindering the characterization of molecular mechanisms for the function of the C‐terminus region by proteomic approaches.^[^ ^11^ ^]^

We wondered whether AlphaFold2 might aid in overcoming these obstacles in elucidating the molecular basis of the C‐terminus variation of GmSWEET10a.^[^ ^3^ ^]^ According to AlphaFold2 prediction, the C‐terminus in the LO‐type forms a long tail with a β‐sheet via multiple hydrogen bonds and “blocks” the protein cavity (Figure 1C). In contrast, the HO‐type exhibits a short C‐tail “outdoors” of the cavity (Figure 1D). Subsequently, we used meta‐dynamics simulations to investigate the free‐energy barriers associated with the release of the tail from the substrate‐transport tunnel of GmSWEET10a LO‐type proteins in their inward‐facing state.^[^ ^16^ ^]^ A barrier of ≈3.0 kcal mol^−1^ was determined for this tail release for both GmSWEET10a LO types, a comparable energy barrier as the substrate‐transport process in other SWEET proteins,^[^ ^16^ ^]^ suggesting that the C‐terminal tail is stable within the tunnel (Figure S4, Supporting Information). We conducted HOLE analysis to visualize the tunnel radius for GmSWEET10a LO‐ and HO‐type transporters.^[^ ^17^ ^]^ The LO‐type GmSWEET10a has narrower tunnels with average radii ≈2 Å, which allows low substrate transport (Figure 1C). For the HO‐type GmSWEET10a, the hole profile consistently shows open cavities, with an average tunnel radius of 4 Å on the intracellular side (Figure 1D). These results suggest that compared with LO‐type GmSWEET10a, the HO‐type GmSWEET10a has a higher likelihood of facilitating sugar transport, which may be associated with increased transport activity.

### 2.2 AlphaFold‐Guided Gene Editing of GmSWEET10a/b to Manipulate Soybean Oil Content

**Figure 2:**
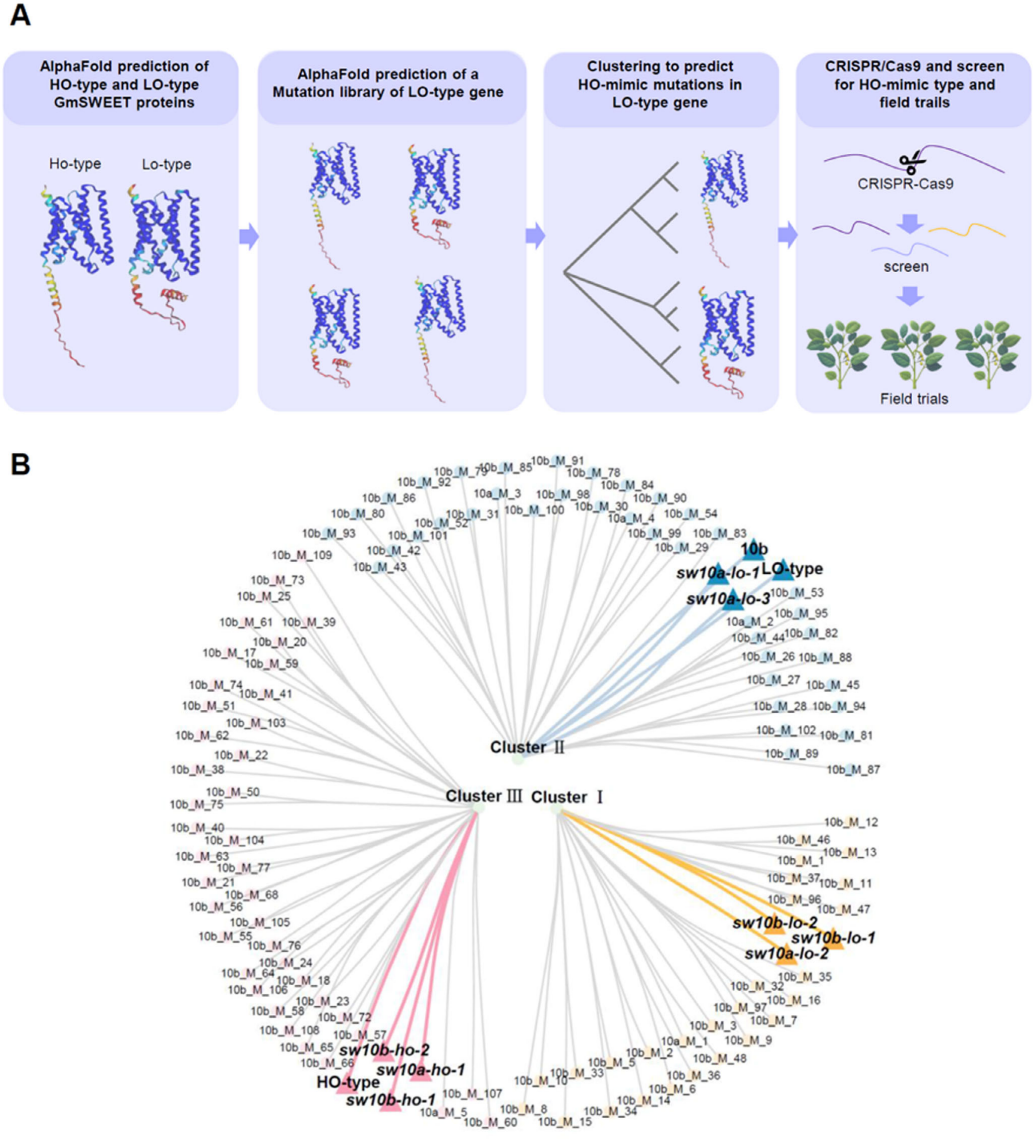
AlphaFold2‐guided bespoke gene editing of GmSWEETs A) Workflow for AlphaFold2‐guided gene editing of soybean SWEET genes toward lifting oil content in seed. B) Protein‐structure‐based clusters of GmSWEET10a/b mutants and their haplotypes. HO‐type GmSWEET10a and GmSWEET10b mutants were grouped into Cluster III, while LO‐type GmSWEET10a and GmSWEET10b mutants were classified into Cluster II or Cluster I. Clusters I, II, and III were marked with yellow, blue, and pink respectively. Mutants or genotypes with functional evidence are labeled with triangles and highlighted. The clustering of different GmSWEET10a/b genotypes and mutants was performed according to the protein structures predicted by AlphaFold2. HO‐type: HO‐type GmSWEET10a; LO‐type: LO‐type GmSWEET10a; M: mutant; 10a: GmSWEET10a; 10b: GmSWEET10b. All protein sequences used for clustering are provided in Table S1 (Supporting Information).

We then developed an AlphaFold‐assisted gene‐editing procedure to help predict C‐terminus mutations of *SWEET* genes that confer improved transport activity (**Figure** **2**A). First, we hypothetically or experimentally generated a series of mutations and predicted the corresponding structures with AlphaFold2. Second, multiple structural alignments were performed with these sequences so we could identify mutation types that cluster closely with HO‐type rather than LO‐type GmSWEET10a isoforms, which exhibit an open cavity and presumably higher transport activity (designated HO‐mimic haplotypes) (Figure 2).

**Figure 3:**
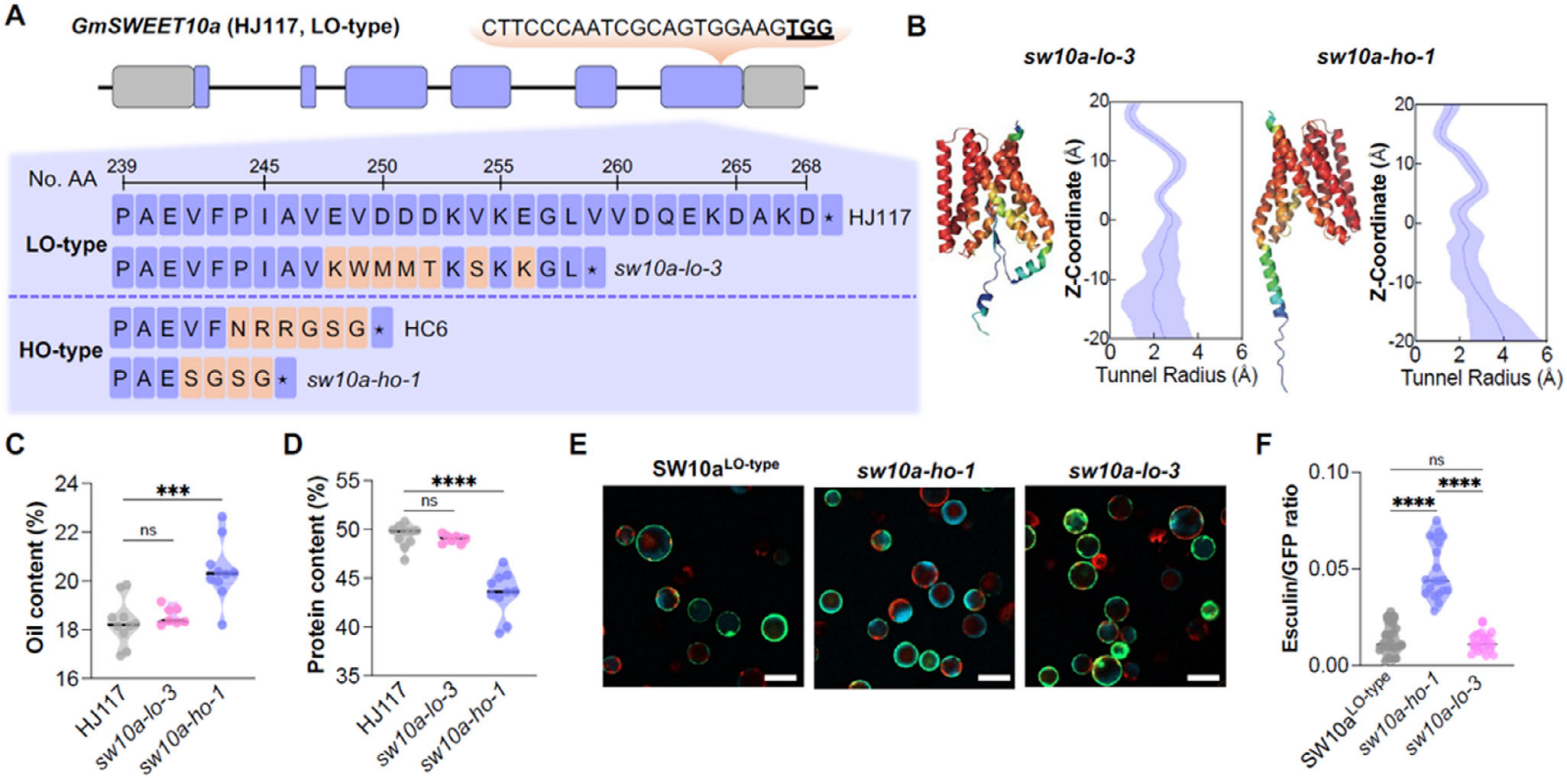
AlphaFold‐assisted gene editing of *GmSWEET10a* improves soybean oil content. A) *GmSWEET10a* gene structure (top) and predicted amino‐acid sequences (bottom) of CRISPR–Cas9 mutants. B) AlphaFold2‐based protein haplotype‐structure and tunnel profile of sw10a‐lo‐3 and sw10a‐ho‐1 GmSWEET10a isoforms. C,D) Quantification of oil content C), and protein content D) for greenhouse‐grown low‐oil wild‐type HJ117 and mutants developed in this background (*n* = 7–10). E) Representative images of Arabidopsis protoplasts transformed with *GmSWEET10a* LO‐type control and GmSWEET10a isoforms *sw10a‐ho‐1* and *sw10a‐lo‐3* co‐expressed with *GFP*. GFP fluorescence is shown in green, esculin fluorescence in cyan, and chlorophyll autofluorescence in red. Scale bar = 50 µm. F) Normalized SWEET transport activity by the ratio of esculin fluorescence intensity in the vacuole of protoplasts versus GFP fluorescence in the plasma membrane. Fluorescence intensities were normalized to the radius of the respective protoplast under the assumption of a spherical shape (*n* ≥ 19). Each dot in (C), (D), and (F) represents an independent measurement or plant. Statistically significant differences in (C), (D), and (F) are indicated by different asterisks (ns indicates non‐significant, ^***^ indicates *p* < 0.001, ^****^ indicates *p* < 0.0001, one‐way ANOVA with Kruskal–Wallis test).

As a proof of concept, we edited the GmSWEET10a C‐terminus in c.v. HJ117, a high‐protein germplasm carrying LO‐type *GmSWEET10a*. In T_1_ lines, a 14‐bp deletion clustered with HO‐type *GmSWEET10a*, which we designated HO‐mimic *sw10a‐ho‐1* (–14 bp) (Figure 2B; **Figure** **3**A,B). This line had higher oil and less protein contents compared with HJ117 controls in greenhouse‐grown plants (Figure 3C,D). Another line (*sw10a‐lo‐3*, –1 bp) with mutations clustered with the LO‐type isoform had less or unchanged oil content and 100‐seed weight (Figure 2B; Figure 3A–D; Figure S5, Supporting Information). Esculin‐uptake assays indicated that the sugar‐transport activity of sw10a‐ho‐1 was indeed higher than the LO‐type GmSWEET10a and sw10a‐lo‐3 (Figure 3E,F; Figure S6, Supporting Information). Therefore, AlphaFold‐assisted gene editing indeed helps predict and identify new, artificial alleles for increasing or decreasing soybean oil content.

### 2.3 AlphaFold‐Guided Gene Editing of GmSWEET10b Improves Soybean Oil Contents Without Yield Penalty

**Figure 4:**
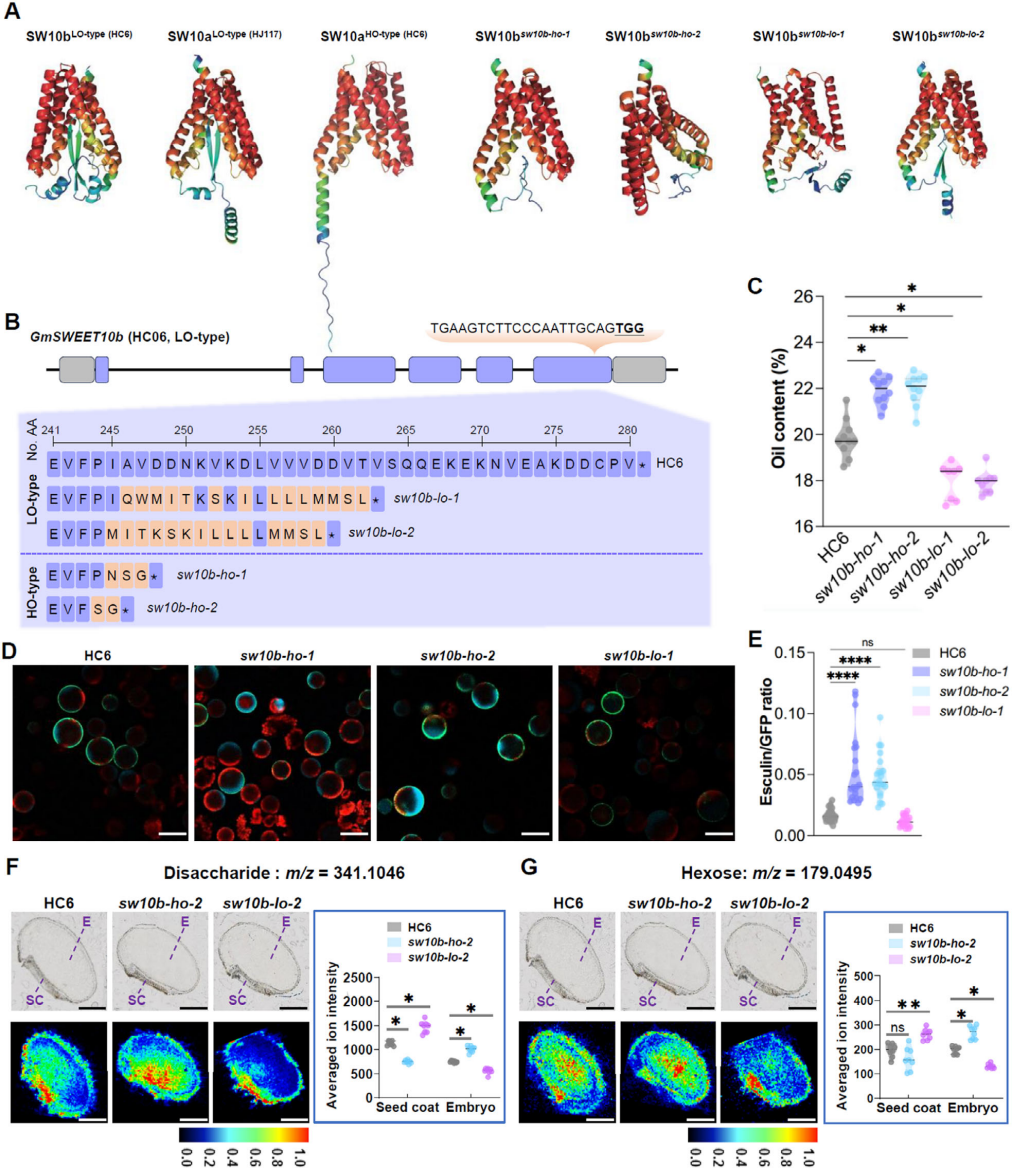
AlphaFold‐informed gene editing of GmSWEET10b lifts soybean sugar transport into embryos. A) AlphaFold2‐based protein haplotype‐structure of LO‐type GmSWEET10b, HO‐type and LO‐type GmSWEET10a isoforms. B) *GmSWEET10b* gene structure (top) and amino‐acid sequences (bottom) of the indicated gene‐edited mutants. C) Oil contents for wild‐type c.v. HC6 and *sw10b* mutants in grown in the greenhouse (*n* = 8–10). D) Representative images of protoplasts in esculin‐uptake assays for GmSWEET10b, sw10b‐ho‐1, sw10b‐ho‐2, and sw10b‐lo‐1 proteins. GFP fluorescence is shown in green, esculin fluorescence in cyan, and chlorophyll autofluorescence in red. Scale bar = 50 µm. E) Quantification of GmSWEET10b transport activity (*n* ≥ 19). F,G) DESI–MSI visualization and averaged ion intensity (n = 8) of spatial distributions of disaccharide (mainly includes sucrose, F) and hexose (mainly includes glucose and fructose, G) in developing seeds of HC6 (WT), *sw10b‐ho‐2* and *sw10b‐lo‐2* mutant plants at 14 days after flowering. E indicates embryo, SC indicates seed coat. Scale bar = 2 mm. Each dot in (C), (E–G) represents an independent measurement or plant. Statistically significant differences in (C), (E–G) are indicated by different asterisks (ns indicates non‐significant, ^*^ indicates *p* < 0.05, ^**^ indicates *p* < 0.01, **** indicates *p* < 0.0001, one‐way ANOVA with Kruskal–Wallis test).

In elite high‐oil cultivars, the HO‐type *GmSWEET10a* haplotype is mostly fixed, thus limiting prospects for oil‐content improvement by editing this gene.^[^ ^11^, ^12^ ^]^ We, therefore, asked whether the AlphaFold‐guided bespoke gene‐editing method has utility for *GmSWEET10b*, which has no known elite alleles in nature, and its transcript expression parallels that of *GmSWEET10a*.^[^ ^11^ ^]^ Structure prediction and metadynamic simulations indicated that the C‐terminal tail of GmSWEET10b also forms a β‐sheet residing in the cavity, thereby adopting a “blocked” state similar to LO‐type GmSWEET10a (**Figure** **4**A; Figure S7, Supporting Information). We targeted the C‐terminus of *GmSWEET10b* to obtain a series of mutations and clustered the structures of identified and hypothetical mutations with HO‐type SWEET10a, LO‐type GmSWEET10a, and original GmSWEET10b structures. Two mutation types, designated *sw10b‐ho‐1* (–2 bp) and *sw10b‐ho‐2* (–8 bp), were more similar to HO‐type GmSWEET10a than LO‐type GmSWEET10a/b, with apparently open substrate‐translocation tunnels (Figure 4A,B; Figure S7, Supporting Information). We also identified other mutations, namely *sw10b‐lo‐1* (–4 bp) and *sw10b‐lo‐2* (–10 bp) (Figure 4A,B). Greenhouse‐grown *sw10b‐ho‐1* and *sw10b‐ho‐2* plants had increased oil content and unchanged seed yield per plant versus c.v. HC6 (Figure 4C; Figure S8, Supporting Information). By contrast, in *sw10b‐lo‐1 and sw10b‐lo‐2*, oil contents were decreased (Figure 4C).

In esculin‐uptake assays, the HO‐type GmSWEET10b *sw10b‐ho‐1* and *sw10b‐ho‐2* proteins indeed exhibited enhanced sugar‐transport activities compared to GmSWEET10b and *sw10b‐lo‐1* (Figure 4D,E; Figure S9, Supporting Information). Toward validating how modification of GmSWEET10b modulates sugar allocation from the seed coat to the embryo, spatially resolved metabolomics was used to visualize and quantify the distribution of disaccharide (mainly including sucrose) and hexose (mainly including glucose and fructose) sugars in seeds by desorption electrospray ionization–mass spectrometry imaging (DESI–MSI). Notably, disaccharides and hexose in *sw10b‐ho‐2* seed were more distributed to embryos and less abundant in *sw10b‐lo‐2* embryos, compared to c.v. HC6 (Figure 4F,G; Table S2, Supporting Information). As a result, principal component analysis showed that the metabolic profiles within the mutant and HC6 embryos were separated, compared with that in the seed coat (Figure S10; Table S2, Supporting Information). These results confirm that sugar transport into embryos is indeed enhanced by engineering GmSWEET10b through structure‐guided gene editing.

**Figure 5:**
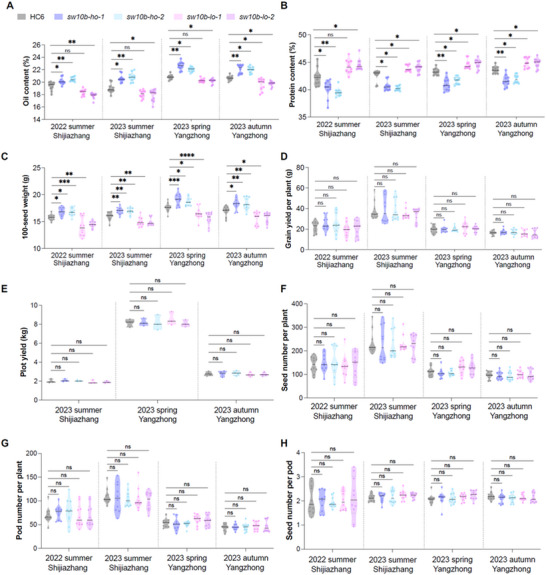
AlphaFold‐informed gene editing of *GmSWEET10b* lifts soybean oil contents in the field without a yield penalty. Quantification of oil content A), protein content B), 100‐seed weight C), grain yield per plant D), plot yield E), seed number per plant F), pod number per plant G), and seed number per pod H) for wild‐type c.v. HC6 and *GmSWEET10b* mutants in multi‐year and multi‐site trials. Each dot represents an independent measurement or plant (*n* = 3–4 for plot yield, *n* = 10 for other traits). Statistically significant differences are indicated by different asterisks (ns indicates non‐significant, ^*^ indicates *p* < 0.05, ^**^ indicates *p* < 0.01, ^***^ indicates *p* < 0.001, ^****^ indicates *p* < 0.0001, one‐way ANOVA with Kruskal–Wallis test for oil and protein content; ordinary one‐way ANOVA with LSD multiple‐comparisons test for other traits).

Multi‐year, multi‐site field trials are required to rigorously evaluate whether a gene modification trait is indeed valuable for breeding.^[^ ^18^ ^]^ We conducted such field trials with wild‐type c.v. HC6, two high‐oil mutants *sw10b‐ho‐1* and *sw10b‐ho‐2*, and two low‐oil mutants *sw10b‐lo‐1* and *sw10b‐lo‐2*, across four seasons and two locations in China: Shijiazhuang (38.03° N, 114.48° E) and Yangzhong (26.28° N, 118.49° E). In all trials, oil contents for *sw10b‐ho‐1* and *sw10b‐ho‐2* were statistically significantly increased by 0.8–1.83 percentage points than that of HC6 seeds (19.35–20.82% across trials) (**Figure** **5**A). Due to a well‐documented negative correlation between soybean oil and protein contents,^[^ ^7^ ^]^ the protein content for HO‐type mutants was decreased by 1.59–2.77 percentage points in all trials than HC6 (42.32–43.62% across trials) (Figure 5B). Meanwhile, LO‐type mutants had a 0.54–1.52 percentage points decrease in oil content, but had 1.21–2.24 percentage points more protein than that of HC6 seeds. In 2023 in Yangzhong, a larger‐scale plot test (30 m^2^ per plot) was performed. *sw10b‐ho‐1* and *sw10b‐ho‐2* showed consistently improved oil content (22.62% in *sw10b‐ho‐1*, 22.04% in *sw10b‐ho‐2* versus 20.82% in HC‐6), and *sw10b‐lo‐1* and *sw10b‐lo‐2* showed consistently improved protein content (44.61% in *sw10b‐lo‐1*, 44.69% in *sw10b‐lo‐2* versus 43.26% in HC‐6). This result demonstrates the potential of AlphaFold‐guided *sw10b* gene editing technology to facilitate the generation of high‐oil or high‐protein soybean varieties.

Most importantly, although the 100‐seed weight of both types of lines changed, grain yield per plant or plot was negligible and without statistical significance (Figure 5C–E). No other statistically significant changes were seen for key agronomic‐performance indicators of plant height, branch number, seed number and pod number per plant, or seed number per pod, except that plant height for *sw10b‐lo‐1* was statistically significantly increased over HC6 in Shijiazhuang in 2023 (Figure 5F–H; Figure S11, Supporting Information). In summary, artificial evolution guided by AlphaFold generated superior *GmSWEET10b* haplotypes with enhanced seed‐oil content without affecting yield performance in the field.

## 3 Discussion

AlphaFold and other machine‐learning tools for predicting 3D protein structure have changed the way protein engineering is done to create novel artificial proteins and redesign natural proteins for improved functionality.^[^ ^4^ ^]^ Here, we demonstrate that gene editing guided by AlphaFold toward HO‐type synthetic alleles for GmSWEET10a/b is of great value for manipulating the oil and protein content in elite soybean cultivars. Such breeding is attained by capitalizing on artificial intelligence's predictive prowess, conducting validation *in planta* via gene editing's bespoke engineering competencies, and undertaking comprehensive field trials, while details regarding precisely how the C‐terminus of SWEET10a/b affects their sugar‐transport activity remain difficult to address.

Improving soybean seed oil and protein contents is difficult due to their complex interrelationships with yield. Protein content is negatively correlated with yield, while oil content is positively related to it. These complex relationships among oil, protein, and yield are hard to adjust and prompt research to understand and manipulate them for better cultivars. Over 30 years, more than 600 genetic regions linked to these traits were identified by QTL analysis. Genes responsible for major seed‐protein QTLs, such as qOil‐15 (*GmSWEET10a*), qOil‐20 (*POWR1*, *Glyma.20G085100*), qOil‐5‐1 (*GmMFT*), and FA9 have been characterized.^[^ ^10^, ^11^, ^12^, ^19^, ^20^, ^21^, ^22^ ^]^ Integrative omics analysis also identified important regulators of soybean oil and protein content, such as *GmRWOS1*.^[^ ^19^ ^]^ Using these variants could be useful for high‐oil soybean cultivars. Nevertheless, many genes with natural variation are strongly selected during domestication and breeding. This, in turn, restricts the potential for exploiting these genetic variations to further enhance seed oil in elite cultivars. As we demonstrated here with GmSWEET10a/10b, with the information around beneficial structural variants, AlphaFold‐guided gene editing may direct the further optimization of oil‐content‐regulating genes and their homologous genes for improved structure and function.

Artificial‐intelligence‐guided protein design may help optimize enzymes toward higher activity for genetic engineering, and such approaches were exploited to guide the design of a modified pectin methylesterase inhibitor that specifically targets and inhibits pectin methylesterases secreted from pathogens but not from plants.^[^ ^23^ ^]^ Nevertheless, AlphaFold may exhibit certain inherent methodological and technical limitations.^[^ ^4^ ^]^ For instance, the current iteration of AlphaFold is unable to predict conformational changes induced by post‐translational modifications or the impact of missense mutations on the 3D structures of proteins.^[^ ^4^ ^]^ In crops, the most abundant genetic variations are SNPs, yet predicting the effect of single amino‐acid changes on protein structure remains an open challenge.^[^ ^9^ ^]^ Therefore, it is desired to advance artificial intelligence tools, such as AlphaMissense, to guide the prediction of structural consequences of missense variants.^[^ ^24^ ^]^

AlphaFold prediction indicated that the C‐terminal tail may block the cavity and affect GmSWEET10a transport activity. In addition, phosphorylation of Arabidopsis SWEET proteins at C‐terminus tails is known, which may affect the conformation and function of SWEETs.^[^ ^25^, ^26^ ^]^ Detailed knowledge of this regulatory mechanism would likely further facilitate the understanding and optimization of SWEET proteins. It is well‐documented that there is a trade‐off between seed oil and protein contents in soybeans.^[^ ^7^ ^]^ Here, the gene‐edited HO‐type *GmSWEET10b* plants had enhanced carbon influx to the seeds and increased oil content, yet accompanied by reductions in protein content. Stacking other beneficial traits to enhance nitrogen availability, such as improving root nodulation,^[^ ^27^ ^]^ may help decouple this negative correlation and increase oil and protein simultaneously.

## 4 Experimental Section

### Plant Material and Growth Conditions

Wild‐type (HC6 and HJ117) and homozygous mutant lines of *GmSWEET10a* and *GmSWEET10b* were used in this study as described in the text. In pot experiments, all soybean plants were cultivated in pots containing nutritional soil (Pindstrup, Denmark) in a greenhouse maintained at 60–80% relative humidity and under a cycle of 14/10 h with temperatures of 28/24 °C (day/night). For field experiments, wild‐type (HC6) and four homozygous mutant lines of *GmSWEET10b* with three or four replicates were planted in Yangzhong (26.28°N, 118.49°E) in Fujian province and Shijiazhuang (38.03°N, 114.48°E) in Hebei province of China from 2022 to 2023 following a randomized block design. On June 28, 2022, soybean seeds were sown in Shijiazhuang, with each plot consisting of one 3‐m‐long row spaced 0.5 m apart. On June 21, 2023, the plot test in Shijiazhuang was designed as three rows wide and 3 m long, with a spacing of 0.5 m, providing an area of ≈4.5 m^2^ for each plot. On April 17 and August 10, 2023, plot experiments in Yangzhong were designed as three rows wide, 25 m and 12.5 m long respectively, with a spacing of 0.4 m, giving an area of ≈30 and 15 m^2^ for each plot. Typical production practices in China were adhered to, with a planting density of 130 000—140 000 plants ha^−1^ in 2022 and 2023. In Shijiazhuang, all plots were fertilized with 375 kg ha^−1^ of compound fertilizer (N:P₂O₅:K₂O = 15:15:15) as base fertilizer. The basic characteristics of the top 20‐cm soils in the field are shown in Table S3 (Supporting Information).

### Plant Trait Investigation

For agronomic trait investigation, once plants attained full maturity (growth stage R8), ≈10 representative individual plants per line were harvested in pot or field experiments. Seven traits were evaluated: plant height, branch number, pod number per plant, seed number per plant, seed number per pod, grain yield per plant, and 100‐seed weight. Approximately 50 seeds per individual plant were used for measuring seed oil and protein contents by employing a MATRIX‐I Fourier‐transform near‐infrared reflectance spectroscope (Bruker Optics) based on a published method.^[^ ^27^ ^]^

### Protein‐Structure Prediction and Clustering

Protein sequences used in this study include GmSWEET10a/b natural haplotypes and 115 hypothetically generated or experimentally identified mutations at C‐terminus of GmSWEET10a/b sequences. Alphafold2 prediction was performed with all the above sequences,^[^ ^3^ ^]^ and protein‐structure diagrams were generated by Pymol (www.pymol.org). Clustering of 118 structures was conducted with the highest average per‐residue confidence metric predicted local‐distance difference test value for each protein structure by the structure‐based clustering algorithm.^[^ ^28^, ^29^ ^]^

### Well‐Tempered Metadynamics Simulation and HOLE Analysis

AlphaFold2‐predicted transporters were protonated using H++ at a pH of 5.3, and they all were inserted into a model plant plasma membrane with 256 lipids, solvated with TIP3P waters, and neutralized with 150 mm KCl using CHARMM–GUI's membrane builder.^[^ ^30^ ^]^ Complex plant plasma‐membrane composition was used to build the lipid bilayer. The systems were each ≈100 000 atoms, with box sizes ≈85 × 85 × 130 Å. For each protein, VMD's membrane mixer was used to create three replicates with 50% lipids being shuffled within their leaflets.^[^ ^31^ ^]^ The systems were minimized using AMBER22's sander for 5000 cycles of steepest descent followed by 45000 cycles of conjugate descent with protein backbone restrained. They were then heated in NVT in five steps to 300 K for a total of 2.5 ns. Systems were then equilibrated in an NPT ensemble with the protein backbone restrained using 5 kcal mol^−1^ Å^2‐1^ for 50 ns, and without restraints for 50 ns. Production runs were 100 ns for each replicate. A Langevin thermostat with a collision frequency of 2.8284 ps^−1^ and a Monte Carlo membrane barostat were used for temperature and pressure regulation. Simulations were run using OpenMM version 8 with the CHARMM36 m force field.^[^ ^32^, ^33^ ^]^ Long‐range electrostatic interactions were calculated using the Particle Mesh Ewald method with a 12 Å cutoff. Free‐energy calculations were performed using OpenMM8 software coupled with the PLUMED 2.7.3 plugin.^[^ ^32^ ^]^

The collective variable (CV) in this simulation was defined as the distance between the center of mass of the β‐sheet region of the tail (residues 231–243) and the center of mass of the top half of the protein. The residues in the top half of the transporter were determined by first calculating the center of mass of the entire transporter and then identifying all atoms located 10 Å above its center. Simulations were performed with a bias deposition frequency of every 500 steps (1 ps), a bias factor of 10.0, and a Gaussian hill height of 0.287 kcal mol^−1^. To ensure efficient sampling, the CV space was divided into a grid ranging from 20 to 65 Å, with 100 bins of size 0.45 Å. A harmonic lower‐wall potential with a force constant of 2.39 kcal mol^−1^ Å^2‐1^ was applied at a CV distance of 17 Å to prevent the system from exploring regions below this threshold. Bootstrapping was done for the estimation of the mean and standard deviation of the free energy profile along the CV. A hole program was used to obtain the tunnel profile for the transporters.^[^ ^17^ ^]^

### Gene Editing of GmSWEET10a/10b by CRISPR–Cas9

CRISPR–Cas9 was performed with previously published tools.^[^ ^34^ ^]^ Guide‐RNA spacer sequences targeting the C‐terminus of *GmSWEET10a/10b* genes were designed, sub‐cloned into pGES201, and introduced into *Agrobacterium tumefaciens* GV3101. Soybean cultivars HC6 and HJ117 were transformed via an *Agrobacterium*‐mediated method as described.^[^ ^34^ ^]^ Guide‐RNA sequences for *GmSWEET10a* and *GmSWEET10b* and primers for genotyping their alleles are listed in Tables S4 and S5 (Supporting Information), respectively.

### RNA Extraction and Quantitative Real‐Time PCR

Total RNA from soybean seed coats was extracted using the E.Z.N.A. RNA Extraction Kit (Omega Bio‐Tek, USA) in accordance with the manufacturer's protocol. The PrimeScript RT Reagent Kit (TaKaRa Biotech, Japan) was used for reverse transcription, and SYBR Premix Ex Taq II ROX Plus Kit (TaKaRa Biotech, Japan) was used for quantitative real‐time polymerase chain reaction amplification. *CYP2* (encoding cyclophilin 2) was employed as a reference gene to calculate the relative expression levels of each gene, as described previously.^[^ ^11^ ^]^ Three biological replicates were used for each sample. Primer sequences are in Table S5 (Supporting Information).

### Protoplast‐Esculin Assay

Protoplasts from Arabidopsis leaves were used to test the uptake activity of SWEET proteins using esculin.^[^ ^13^ ^]^ The vector for *SWEET* expression was modified using pAN580 as the vector backbone.^[^ ^35^ ^]^ A new amplified CaMV *35S* promoter was ligated onto the pAN580 vector, which had been digested with *Sma*I and *Bam*H1, for driving *GFP* expression. Subsequently, the coding sequences of *AtSWEET11*, *GmSWEET10a*, *GmSWEET10b*, or the coding sequences of their mutants were bridged with the *NOS* terminator using overlap PCR and inserted into the modified vector using *Spe*I and *Xba*I restriction sites. Primer sequences used for vector construction are listed in Table S6 (Supporting Information). The original CaMV *35S* promoter in pAN580 was used to drive the expression of *SWEET* genes to enable co‐expression of *SWEET* and *GFP*.

Protoplasts were obtained from the leaves of *Arabidopsis thaliana* Col‐0 plants grown under long‐day conditions for three weeks and were transformed using previously described methods.^[^ ^36^, ^37^ ^]^ Esculin was added to the overnight incubated protoplasts at a final concentration of 1 mm. After incubation in the dark for 6 h, an LSM880 confocal laser‐scanning microscope (Carl Zeiss) was used to observe esculin fluorescence within the protoplasts. A 405 nm laser was used to excite esculin, while a 488 nm laser was used to excite GFP and chloroplast autofluorescence. The wavelength range of the detector window was set as previously described.^[^ ^13^ ^]^ To improve consistency in fluorescence comparison, the focus was set so that the plasma membrane appeared as a uniformly wide green ring in the GFP channel. Images of the equatorial plane of these protoplasts were captured. The fluorescence intensities of esculin and GFP, as well as the radius of the cells, were quantified using ZEN 3.10 software (Carl Zeiss). The fluorescence intensity of GFP was used to represent the expression level of SWEET protein. The efficiency of esculin uptake by SWEET in each cell was calculated using the formula:

(1) $$\mathrm{Ratio}=\frac{\mathrm{Esculin}\;\mathrm{fluorescence}\;\mathrm{intensity}/R^{3}}{\mathrm{GFP}\;\mathrm{fluorescence}\;\mathrm{intensity}/R^{2}}$$

### DESI Imaging

Fresh soybean seeds were embedded with 2.5% carboxymethyl cellulose in disposable base molds (15 × 15 × 5 mm, Fisherbrand), and flash‐frozen in liquid N_2_. The embedded tissue blocks were transferred to a cryostat (Leica CM1950 S) and allowed to thermally equilibrate at –20 °C for at least 2 h. The frozen sample was then cut into 35‐µm‐thick sections, thaw‐mounted onto Superfrost Plus slides (Fisher Scientific, USA), and vacuum‐dried in a desiccator.^[^ ^38^ ^]^

DESI imaging was performed using a SYNAPT XS mass spectrometer coupled to a DESI‐XS ion source (Waters, United Kingdom). The DESI spray solvent was methanol:water:formic acid 98:2:0.1 (% v/v/v), delivered with a flow rate of 3 µL min^−1^. The capillary voltage was 0.5 kV, and the nitrogen gas pressure was 0.5 bar. A heated transfer line was used at 255 °C to improve ionization and transmission of the molecules into the mass spectrometer. The pixel size of X and was set to 100 µm, and the grating speed at 400 µm s^−1^. DESI imaging data were acquired in full‐scan mode in positive ion mode with a sampling cone voltage of 40 V and a scanning range of 50 –1500 Da.

MassLynx Software V4.2 (Waters Corporation) was used for data acquisition and spectrum preview. HDI v1.6 software was used to export the data into imzML format (Waters, United Kingdom). The imzML‐format DESI–MSI data were imported into ShinyCardinal V0.3.5^[^ ^39^ ^]^ to perform data preprocessing and visualization. The regions of interest were manually defined in ShinyCardinal, and the extracted ion intensities were used for further statistical calculations.

### Statistical Analysis

Phenotypic analysis and figure generation for the agronomic and seed‐composition traits were performed with GraphPad Prism (version 9.0). For esculin/GFP ratios, seed oil, and protein contents, all data were compared using Mann–Whitney tests or one‐way ANOVA with Kruskall–Wallis tests and multiple‐comparisons tests by the two‐stage linear step‐up procedure.^[^ ^40^ ^]^ For other traits, all data were compared using two‐tailed Student's *t*‐tests or ordinary one‐way ANOVA with an LSD multiple‐comparison test. ^*^ *p* < 0.05, ^**^ *p* < 0.01, ^***^ *p* < 0.001, ^****^ *p* < 0.0001.

## Conflict of Interest

GZU as the applicant has filed patent applications in both China (Application No. 202510406505.6) and through the PCT route (Application No. PCT/CN2025/086583), with Y.G., J.W., M.B., and H.K., as co‐inventors (All co‐inventors are authors in the manuscript). The patent covered the applications of mutation of the *GmSWEET10a/b* gene to improve soybean oil content. The remaining authors declare no competing interests.

## Author Contributions

J.W., L.Z., S.W., and X.W. contributed equally to this work and performed most experiments, and analysed data. L.Y., X.S., S.Z., J.S., M.L., and X.J.W. contributed to field trials. S.L., P.G., M.Y., and Z.Z. generated *sweet* mutants. M.B. prepared figures. H.K. performed soybean transformation. A.P., N.T., and D.S. performed the simulations and analyzed the results. Y.D. and Z.D. contributed to the DESI imaging. Y.G. designed the experiments, wrote the paper together with other authors, and conducted project administration and funding acquisition.

## Supporting information



Supporting Information

Supplemental Table 1

Supplemental Table 2

Supplemental Table 3

Supplemental Table 4

Supplemental Table 5

Supplemental Table 6

## Data Availability

Gene‐editing lines and plasmids generated are available upon request from the corresponding authors, in compliance with the regulatory policy on gene‐edited crops and soybean germplasm from the Ministry of Agriculture and Rural Affairs of the People's Republic of China (MOA). Source data are provided along with this paper.
