# Supplementary material for: AlphaFold‐Guided Bespoke Gene Editing Enhances Field‐Grown Soybean Oil Contents
Source: Adv Sci (Weinh). 2025 May 14;12(23):2500290. doi: 10.1002/advs.202500290 (PMC12199397; doi:10.1002/advs.202500290)
Supplement: Supplementary file 1 — Supporting Information [file ADVS-12-2500290-s006.docx]

Supporting Information

AlphaFold-guided bespoke gene editing enhances field-grown soybean oil contents

Jie Wang, Li Zhang, Shoudong Wang, Xin Wang, Suning Li, Pingping Gong, Mengyan Bai, Arnav Paul, Nathan Tvedt, Hengrui Ren, Maoxiang Yang, Zhihui Zhang, Shaodong Zhou, Jiayi Sun, Xianjin Wu, Huaqin Kuang, Zhenghua Du, Yonghui Dong, Xiaolei Shi, Meina Li, Diwakar Shukla, Long Yan and Yuefeng Guan*


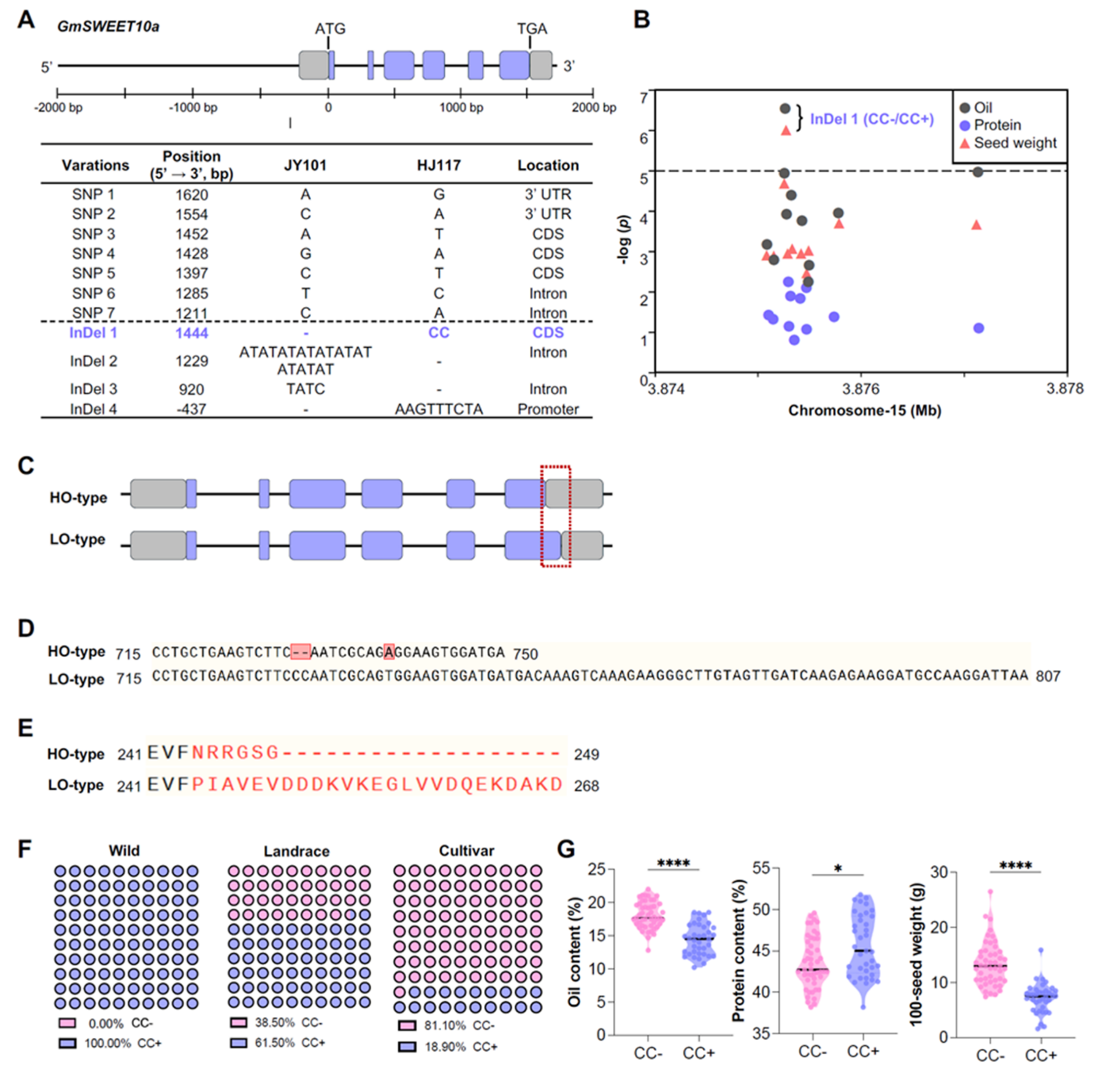


**Figure S1. InDel 1 (CC–/CC+) is most statistically significantly associated with oil content.** **(A)** *GmSWEET10a* sequence comparison between HJ117 and JY101. **(B)** Regional association analysis identified InDel 1 (CC–/CC+) as most statistically significantly associated with oil content using a general linear model. Genotypic values for each trait are from SoyBase (https://www.soybase.org) and SNP and indel data are from SoyKB (http://soykb.org/). **(C)** Schematic of *GmSWEET10a* sequence variation in exon 6 between HO-type and LO-type. The location of InDel 1 (CC–/CC+) causing a sequence difference is enclosed within a red rectangle. **(D, E)** The CC deletion in HO-type *GmSWEET10a* causes a frameshift **(D)** and a truncated protein at the C-terminus **(E)**. **(F)** Frequency distribution of InDel 1 (CC–/CC+) genotypes amongst sequenced wild soybeans (n = 3), landraces (n = 39) and improved cultivars (n = 53). **(G)** Comparison of oil content, protein content, and 100-seed weight among different InDel 1 genotypes. The number of samples in each haplotype (n) is (CC–) = 58 and (CC+) = 37. Each dot represents an independent haplotype. * indicates *P* < 0.05, **** indicates *P* < 0.0001 (Mann–Whitney test for seed oil and protein contents; two-tailed Student’s t-test for 100-seed weight).


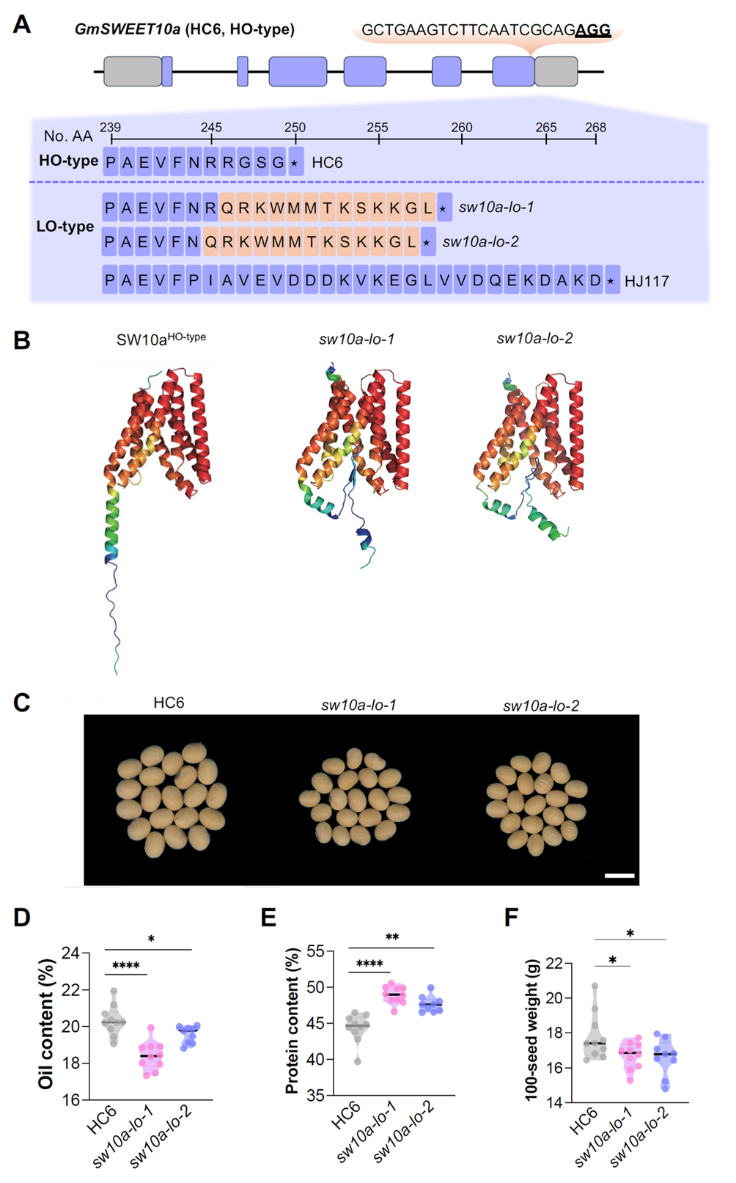


**Figure S2. Gene editing the C-terminus of HO-type GmSWEET10a in HC6 decreases soybean oil content.** **(A)** *GmSWEET10a* gene structure (top) and predicted amino-acid sequences (bottom) of CRISPR–Cas9 mutants. **(B)** AlphaFold2-based protein haplotype-structure of sw10a-lo-1 and sw10a-lo-2 isoforms of GmSWEET10a. **(C)** Seed appearance for wild-type c.v. HC-6 and *sweet10a* mutants (Scale bar = 1 cm). **(D–F)** Oil content **(D)**, protein content **(E)**, and 100-seed weight **(F)** of the wild-type c.v. HC-6 and *sweet10a* mutants grown in the greenhouse. Each dot represents an independent measurement or plant (n = 9–10). Statistically significant differences are indicated by different asterisks (* indicates *P* < 0.05, ** indicates *P* < 0.01, **** indicates *P* < 0.0001, one-way ANOVA with Kruskal–Wallis test for oil and protein content; ordinary one-way ANOVA with LSD multiple-comparisons test for 100-seed weight).


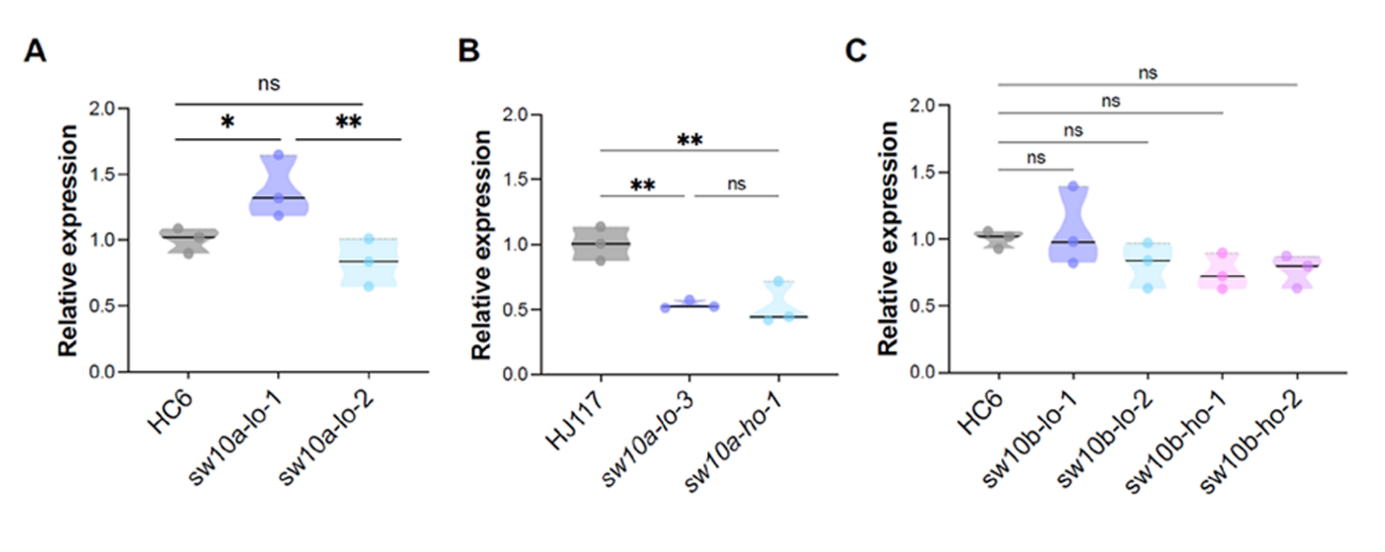


**Figure S3. qRT–PCR analyses of *GmSWEET10a/b* expression in the seed coat at 28 days after flowering. (A)** *GmSWEET10a* expression in *sweet10a* mutants in the wild-type c.v. HC6 background. **(B)** *GmSWEET10a* expression in *sweet10a* mutants in the wild-type c.v. HJ117 background. **(C)** *GmSWEET10b* expression in *sweet10b* mutants in the wild-type c.v. HC6 background. Each dot represents an independent biological replicate (n = 3) and relative expression was normalized to *GmCYP2*. Statistically significant differences are indicated by different asterisks (ns indicates non-significant, * indicates *P* < 0.05, ** indicates *P* < 0.01, one-way ANOVA with LSD multiple comparisons test).


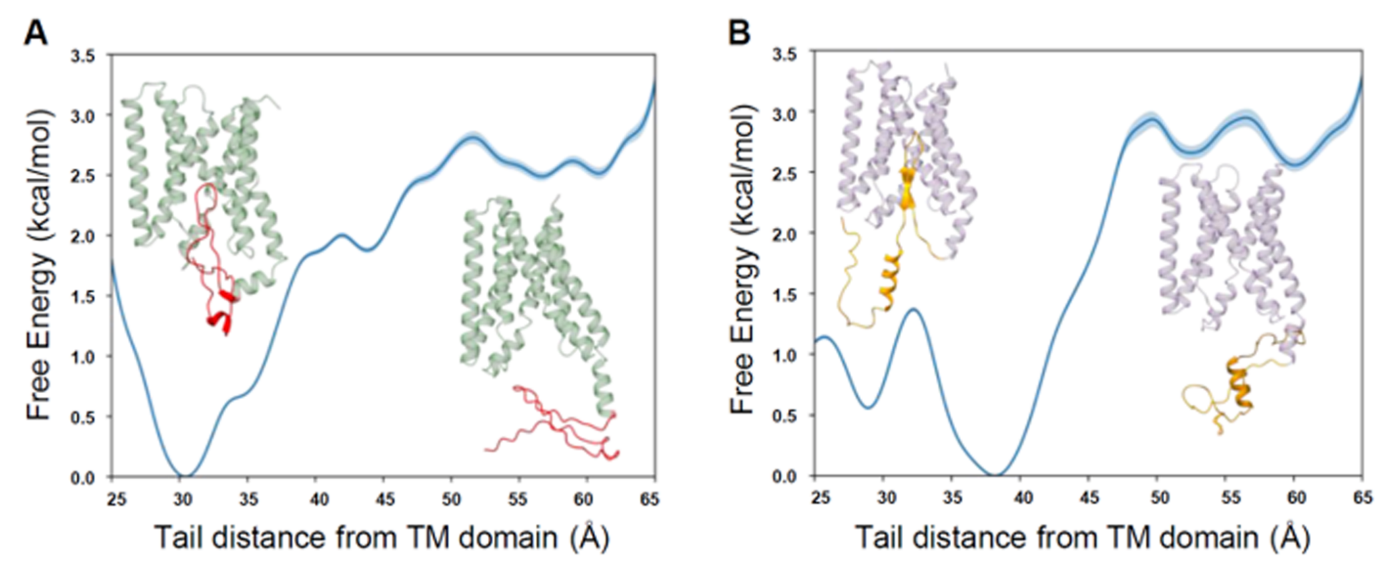


**Figure S4. Free-energy profiles for tail release from the GmSWEET10** **transmembrane (TM) domains. (A)** GmSWEET10a LO type and **(B)** GmSWEET10b representative structures for tail in and tail out are shown.

**
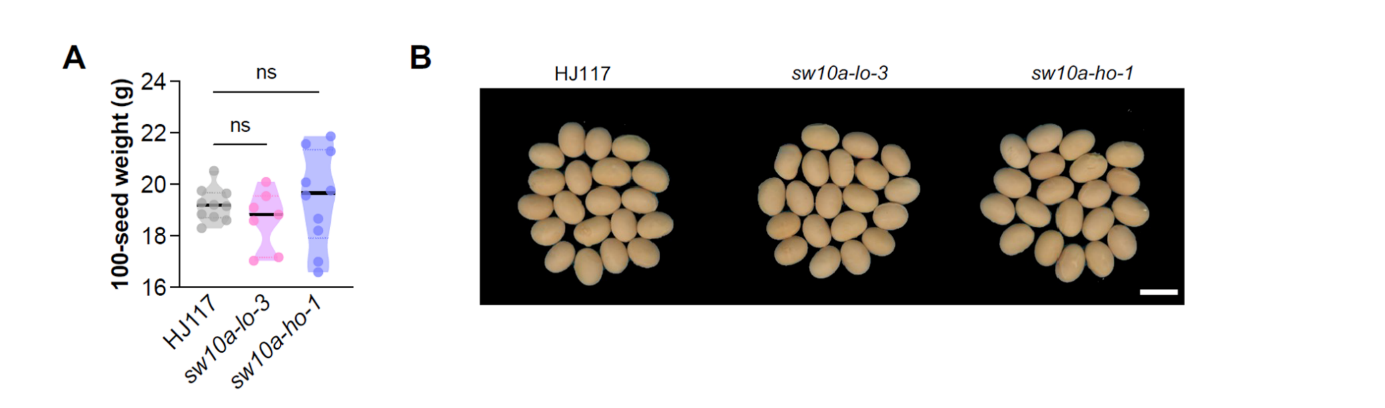
**

**Figure S5. Gene editing the C-terminus of LO-type GmSWEET10a in HJ117 has no obvious effect on 100-seed weight** **for greenhouse-grown plants.** Each dot in **A** represents an independent measurement or plant (n = 7–10). One-way ANOVA with LD multiple comparisons test were performed in **A** (ns indicates non-significant). Scale bar = 1 cm.

**
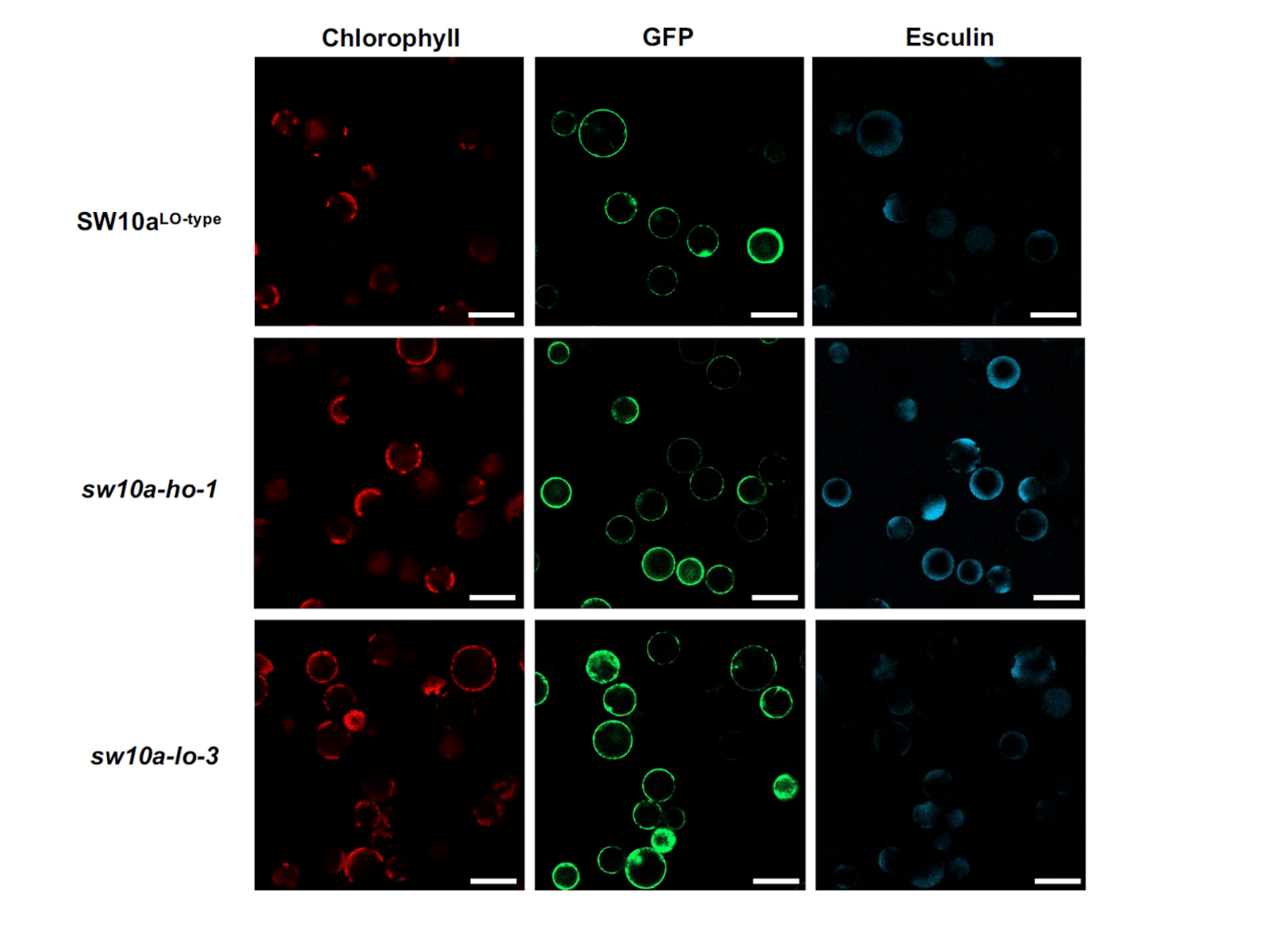
Figure S6. Sugar-transport activity of sw10a-ho-1 is higher than the LO-type GmSWEET10a and sw10a-lo-3.** Separate, representative images of Arabidopsis protoplasts transiently transformed with *GmSWEET10a* LO-type (wild-type *GmSWEET10a* from c.v. HJ117), *sw10a-ho-1* and *sw10a-lo-3* genotypes co-expressed with *GFP*. GFP fluorescence is shown in green, esculin fluorescence in cyan and chlorophyll autofluorescence in red. Scale bar = 50 μm.

**
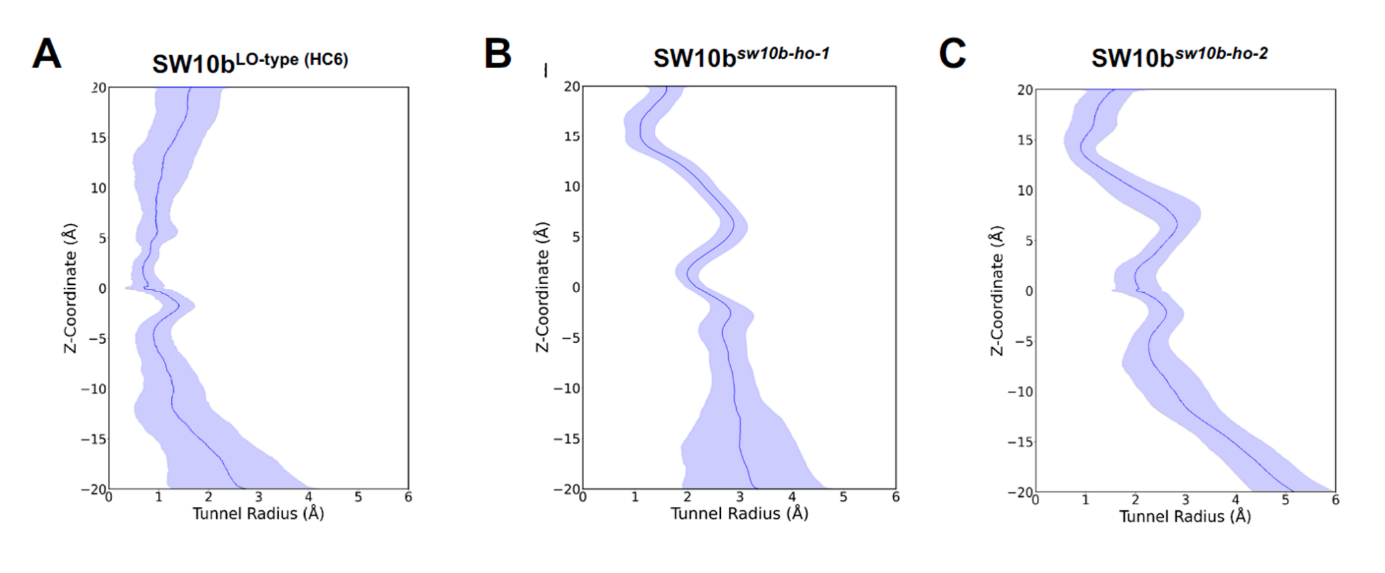
Figure S7. Tunnel profiles for GmSWEET10b and HO mutants. (A)** GmSWEET10b **(B)** *sw10b-ho-1* and **(C)** *sw10b-ho-2.* Shaded areas show the standard deviation of the tunnel profile.

**
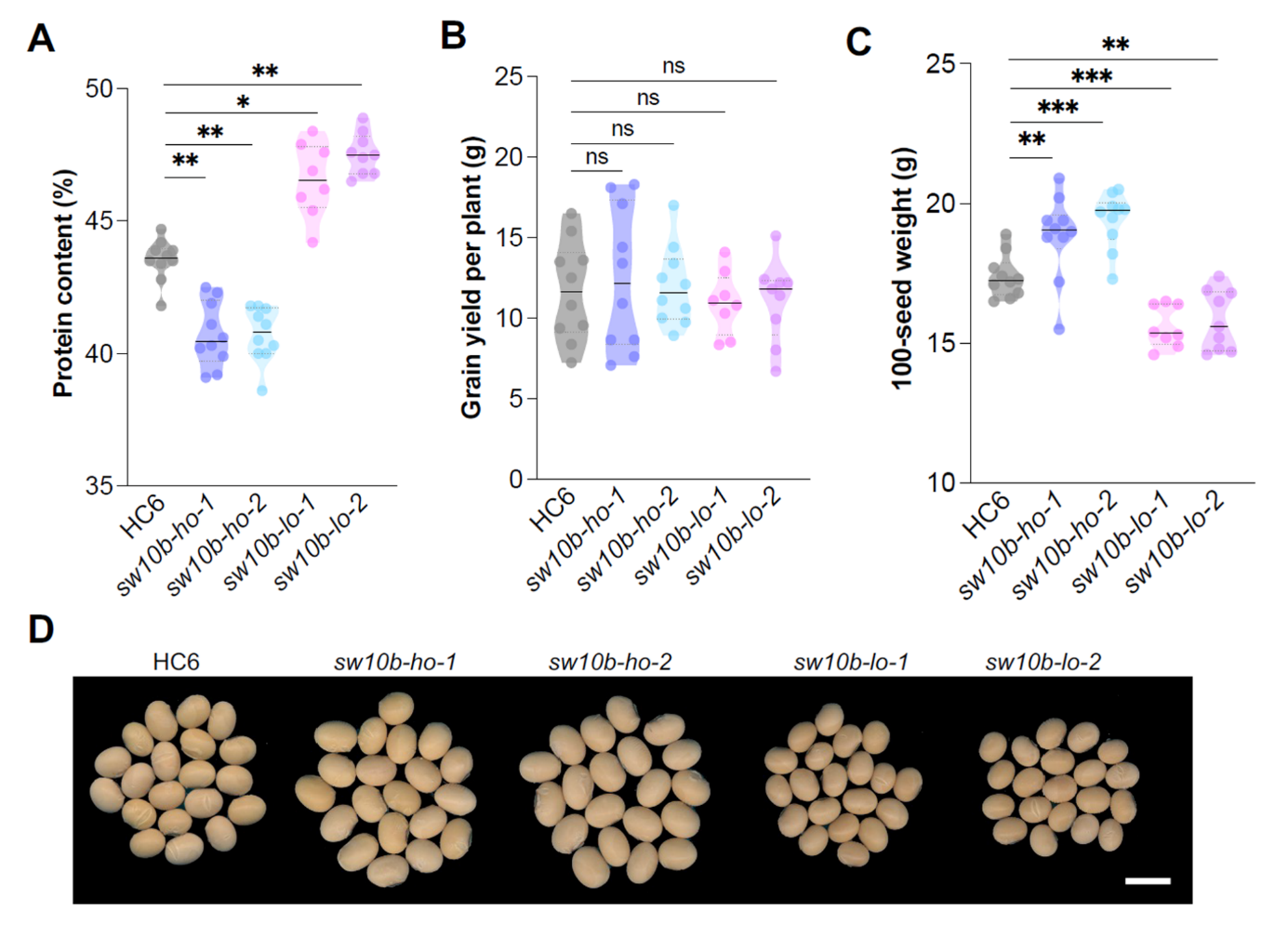
Figure S8. Artificial GmSWEET10b alleles grown in the greenhouse have defects in protein content (A), 100-seed weight (C,D) and no defects in grain-yield per plant (B).** Each dot in **A–C** represents an independent measurement or plant (n = 10). Statistically significant differences in **A–C** are indicated by different asterisks (ns indicates non-significant, * indicates *P* < 0.05, ** indicates *P* < 0.01, *** indicates *P* < 0.001, one-way ANOVA with Kruskal–Wallis test for protein content; ordinary one-way ANOVA with LSD multiple-comparisons test for 100-seed weight and grain yield per plant). The scale bar in **D** is 1 cm.

**
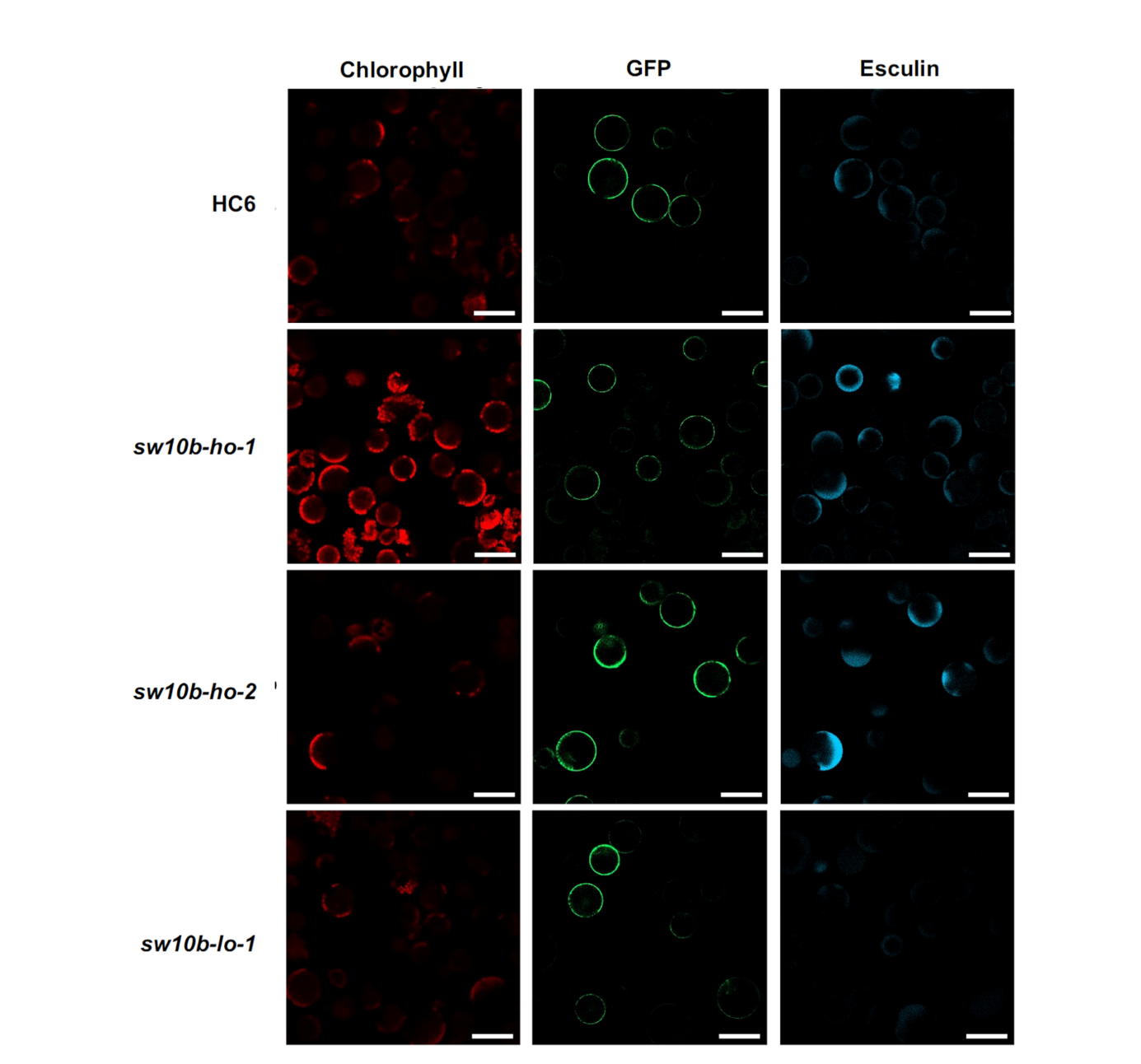
Figure S9. HO-type sw10b-ho-1 and sw10b-ho-2 proteins have enhanced sugar-transport activities compared to GmSWEET10b and sw10b-lo-1.** Separate, representative images of Arabidopsis protoplasts transformed with wild-type *GmSWEET10b* (HC6), *sw10b-ho-1, sw10b-ho-2, sw10b-lo-1,* co-expressed with *GFP*. GFP fluorescence is shown in green, esculin fluorescence in cyan and chlorophyll autofluorescence in red. Scale bar = 50 μm.

**
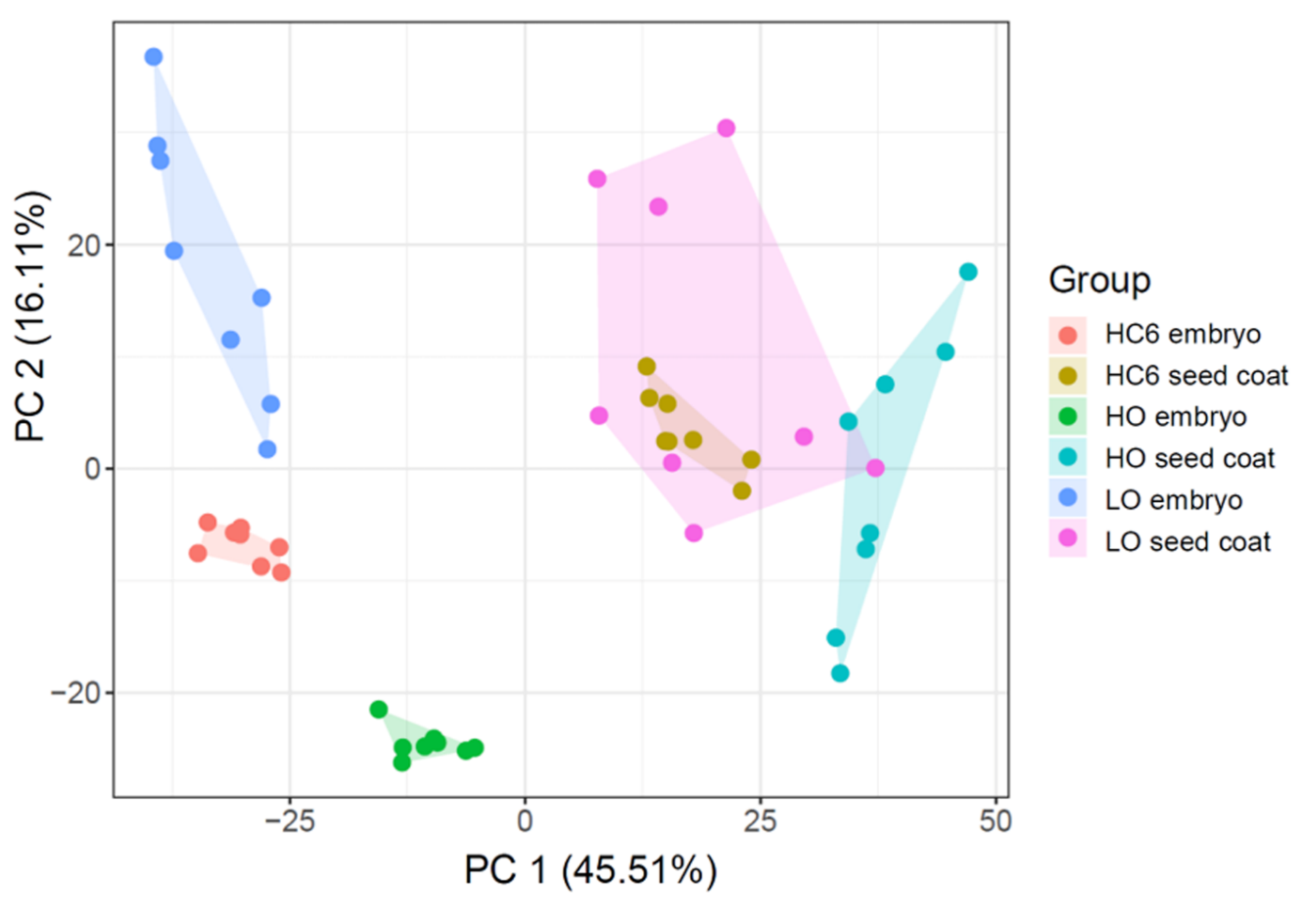
Figure S10. Principal-component analysis of metabolic data for all samples (n = 8).** ‘HO’ and ‘LO’ indicates *sw10b-ho-2* and *sw10b-lo-2*, respectively.

**
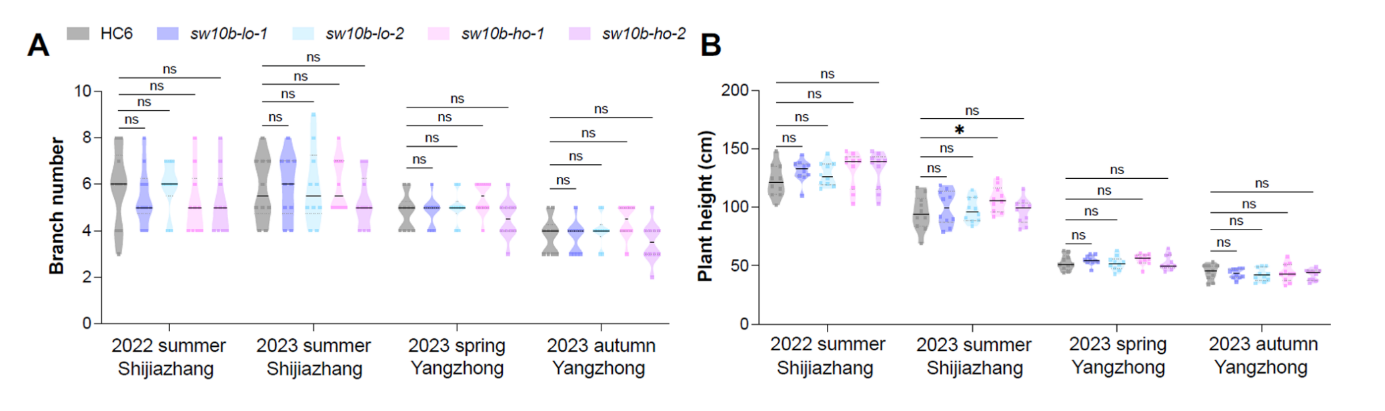
Figure S11. Artificial *GmSWEET10b* alleles grown in the field have no or minor defects in agronomic performance.** Branch number (**A**) and plant height (**B**) for wild-type HC6 and *sweet10b* mutants in multi-year and multi-site field trials. Each dot represents an independent measurement or plant (n = 10). Statistically significant differences are indicated by different asterisks (ns indicates non-significant, * indicates *P* < 0.05, ordinary one-way ANOVA with LSD multiple-comparisons test).
